# The Slow Growth of Adventitious Roots in Tetraploid Hybrid Poplar (*Populus simonii* × *P. nigra* var. *italica*) May Be Caused by Endogenous Hormone-Mediated Meristem Shortening

**DOI:** 10.3390/plants13111430

**Published:** 2024-05-22

**Authors:** Lixia Wu, Yuxin Ren, Xuefang Wang, Yuntong Zhang, Jun Wang

**Affiliations:** 1 State Key Laboratory of Tree Genetics and Breeding, Beijing Forestry University, Beijing 100083, China; wlx17584546080@163.com (L.W.); ryx18236330386yyy@163.com (Y.R.); xuefangwangjxau@163.com (X.W.); canon534534@163.com (Y.Z.); 2 National Engineering Research Center of Tree Breeding and Ecological Restoration, Beijing Forestry University, Beijing 100083, China; 3 Key Laboratory of Genetics and Breeding in Forest Trees and Ornamental Plants, Ministry of Education, Beijing Forestry University, Beijing 100083, China; 4 College of Biological Sciences and Technology, Beijing Forestry University, Beijing 100083, China

**Keywords:** adventitious root, meristem, endogenous hormone, poplar, tetraploid

## Abstract

Polyploidization produces abundant phenotypic variation. Little is currently known about adventitious root (AR) development variation due to polyploidization. In this study, we analyzed the morphological, cytological, and physiological variations in AR development between tetraploid and diploid *Populus* plants during in vitro rooting culture. Compared to the diploids, the AR formation times and rooting rates of the tetraploids’ stem explants had non-significant changes. However, the tetraploid ARs exhibited significantly slower elongation growth than the diploid ARs. Cytological observation showed that the tetraploid ARs were characterized by shorter root meristems and reduced meristem cell numbers, suggesting the reasons for the slow AR elongation. Analysis of hormones and related metabolites during AR development demonstrated that the total auxin, cytokinin, and jasmonic acid contents were significantly lower in the tetraploid ARs than in those of the diploids, and that the ratio of total auxins to total CKs at 0 h of AR development was also lower in the tetraploids than in the diploids, whereas the total salicylic acid content of the tetraploids was consistently higher than that of the diploids. qPCR analysis showed that the expression levels of several hormone signaling and cell division-related genes in the tetraploid ARs significantly differed from those in the diploids. In conclusion, the slow elongation of the tetraploid ARs may be caused by the endogenous hormone-mediated meristem shortening. Our findings enhance the understanding of polyploidization-induced variation in AR development of forest trees.

## 1. Introduction

Polyploids, which are organisms with three or more complete sets of chromosomes, are widespread among angiosperms [1,2,3]. Many previous studies have shown that polyploidization leads to considerable changes in the morphology, physiology, and biochemistry of plants and is a key driver of macro-evolutionary success [3,4,5,6]. Specifically, polyploidization has been shown to change plants’ height [7,8,9], leaf index [10], fruit size [11,12], and secondary metabolite levels [13,14], among others.

Roots are vital organs of plants, and a well-developed root system improves water and nutrient uptake efficiencies by exploring a larger volume of soil [15,16,17]. However, the effects of polyploidization on root growth vary among species, with some promoting growth [18,19], and others suppressing it [20,21], resulting in long or short root phenotypes, respectively. In woody plants, tetraploid plants of *Citrus wilsonii* [22], *Malus prunifolia* [23], and Carrizo Citrange [20] have shown slow adventitious root (AR) growth. Although these polyploids demonstrate high drought stress resistance, salt stress resistance, or boron excess tolerance, slow root growth might reduce the efficiency of asexual reproduction and restrict their application in plantations. The reasons behind the retardation in AR growth of polyploid trees are currently unknown.

The growth and the development of roots are maintained by the root apical meristem (RAM) [24], and the length of the RAM is proportional to the root length [25,26,27]. Furthermore, the length of the RAM is affected by polyploidization. Diploid hybrid sweetgum (*Liquidambar styraciflua* × *L. formosana*) still showed active RAM cell division during the late stage of rooting culturing, while tetraploid roots exhibited a shorter RAM length, reduced longitudinal elongation ability, and significantly shorter root length than those of diploid sweetgum [28]. Previous studies have reported that RAM development and root growth are affected by phytohormones [24], and the levels of certain hormones in plants are affected by the ploidy levels. More indole-acetic acid, active gibberellin (GA), salicylic acid (SA), and active cytokinin (CK) was detected in the roots of autotetraploid *Salix viminalis* [29]. The levels of 6-BA in tetraploid *Liriodendron sino-americanum* leaves were significantly different from those of diploids [30]. Levels of GA and the endogenous auxin brassinolide were significantly lower in tetraploid sweetgum roots and stems than in those of diploids [28]. Therefore, it is important to analyze the changes in hormone levels of polyploid roots in order to understand the variation in root growth traits. However, how endogenous hormone levels change during polyploid root development to regulate root meristem size, leading to variation in root growth, has not yet been elucidated.

Poplar is well known as a woody model plant because of its rapid growth, large biomass, simplicity of asexual reproduction, and efficient breeding and transformation possibilities [31,32,33]. Slow growth of ARs has been observed in tetraploid poplars [34], but the reasons behind the variation are not clear. To investigate the effect of ploidy variation on AR development, this study observed the differences in AR morphology between diploid and autotetraploid poplars cultured in vitro at different growth stages. Morphological, cytological, and physiological levels were combined to analyze the development variation in the ARs of tetraploid poplars. The purpose of this study is to clarify whether the slow growth of tetraploid ARs is related to the size of the meristem, and more importantly, to analyze how hormones and related metabolites (HRMs) affect the size of the root meristem to potentially cause slow growth of tetraploid ARs. The results provide a basis for further exploration of the mechanism behind the slow growth of ARs in tetraploid trees.

## 2. Results

### 2.1. Comparison of the Development of ARs between Diploids and Tetraploids

**Figure 1:**
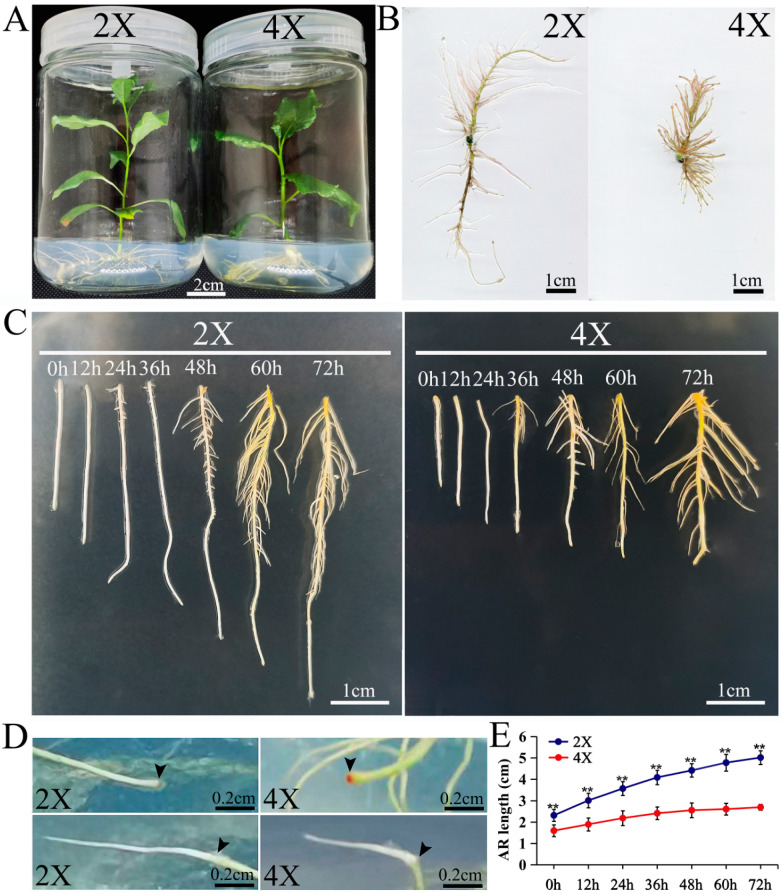
Morphological phenotypes at different time points of AR development in diploid and tetraploid poplars. (**A**) Photos of 2X and 4X one-month-old seedlings were used in this study. (**B**) AR phenotype of 2X and 4X growth at one month. (**C**) Close-up views of 2X and 4X ARs at seven time points. (**D**) The photographs depict 2X and 4X ARs with reddened root tips (**above**) and ARs that continued to elongate from the red root tip position (**below**). The red coloration of the AR tip, indicated by the arrow above, signifies the cessation of elongation. Conversely, in the figure below, the root resumed elongating from the red root tip position. (**E**) AR lengths at different time points in 2X (diploid) and 4X (tetraploid). Three independent experiments were performed (n = 15 for each experiment) in (**E**). Data are represented by means ± SD. ** *p* < 0.01 (Student’s *t*-test).

There were significant trait differences between the diploid and autotetraploid poplars cultivated for one month (Figure 1A), especially in the adventitious root length (ARL), which varied considerably, with average root lengths of 6.06 cm for the diploids and 3.49 cm for the tetraploids (Figure 1B). To characterize the entire AR development processes of the diploids and tetraploids, a time-course experiment was performed on the third day of AR formation, and the ARL was measured every 12 h. During AR development, it was found that the ARs no longer elongated after 72 h, and the root tips gradually turned redder over time (Figure 1D). After a period of AR thickening, the root tips resumed elongation growth (Figure 1D). Therefore, the AR morphology was characterized (Figure 1C), and the corresponding AR lengths (Figure 1E) were measured at seven time points (0 h, 12 h, 24 h, 36 h, 48 h, 60 h, and 72 h). The ARL continuously increased from 0 h (2.32 and 1.60 cm in the diploids and tetraploids, respectively) to 72 h (5.02 and 2.69 cm in the diploids and tetraploids, respectively). The ARLs of the diploids and the tetraploids were significantly different (*p* < 0.05) at all the time points (Figure 1C,E). The elongation rate of the diploid ARs was higher than that of the tetraploid ARs, with those of the diploids and tetraploids increasing by 2.70 cm and 1.09 cm from 0 h to 72 h, respectively, and both significantly decreased at 36 h (Figure 1E). These data suggest that genome doubling inhibits the elongation of ARs.

### 2.2. Comparison of AR Formation Capacities of Diploids and Tetraploids

**Figure 2:**
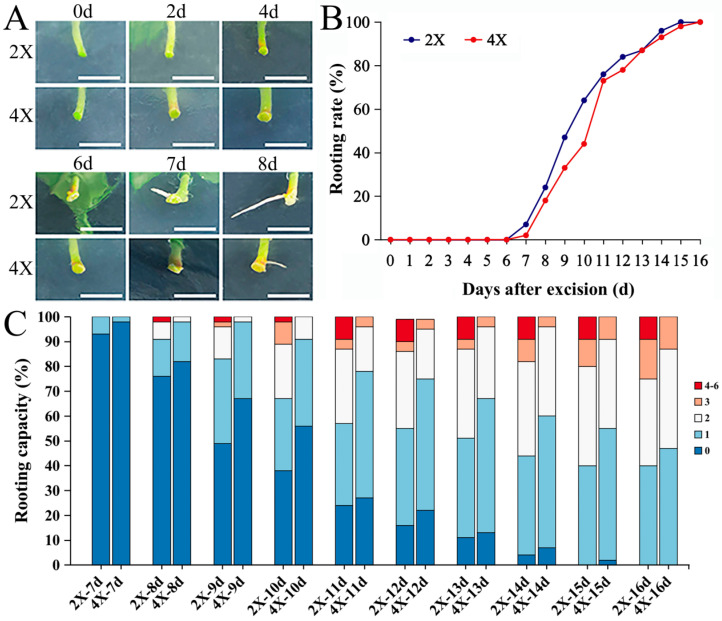
Analysis of AR formation ability of diploid and tetraploid poplars. (**A**) Comparison of various rooting stages of micro-cutting between 2X and 4X poplars. Bar = 1 cm. (**B**) Rooting rate over 16 days following micro-cutting of 2X and 4X. (**C**) Comparison of rooting capacities of 2X and 4X over time. Three independent experiments were performed (n = 15 for each experiment), as shown in (**A**–**C**), on 2X, diploid, and 4X, tetraploid.

To determine whether the AR development differences between the diploids and tetraploids were related to AR formation and the rooting rate, we observed them (Figure 2A). During the process of AR formation, the bases of the tetraploids turned red (micro-cutting for two days) one day earlier than those of the diploids, then each base slowly expanded and formed a granular bulge on the seventh day, and the ARs grew from the protrusion position. AR formation began on the seventh day of micro-cutting culturing in both the diploid and tetraploid stem segments, and the difference in rooting time was no more than 12 h (Figure 2A). The rooting rates of the diploids and tetraploids (Figure 2B) did not differ significantly with AR growth. The number of ARs reached a stable level on day 16 of micro-cutting, when the number of ARs was predominantly one or two for most of the diploids and tetraploids, with a few diploids having from four to six ARs (Figure 2C). The above results suggest that the slow AR growth of tetraploids is not influenced by the time of AR formation or the rooting rate.

### 2.3. Histological Observation of ARs of Diploids and Tetraploids

**Figure 3:**
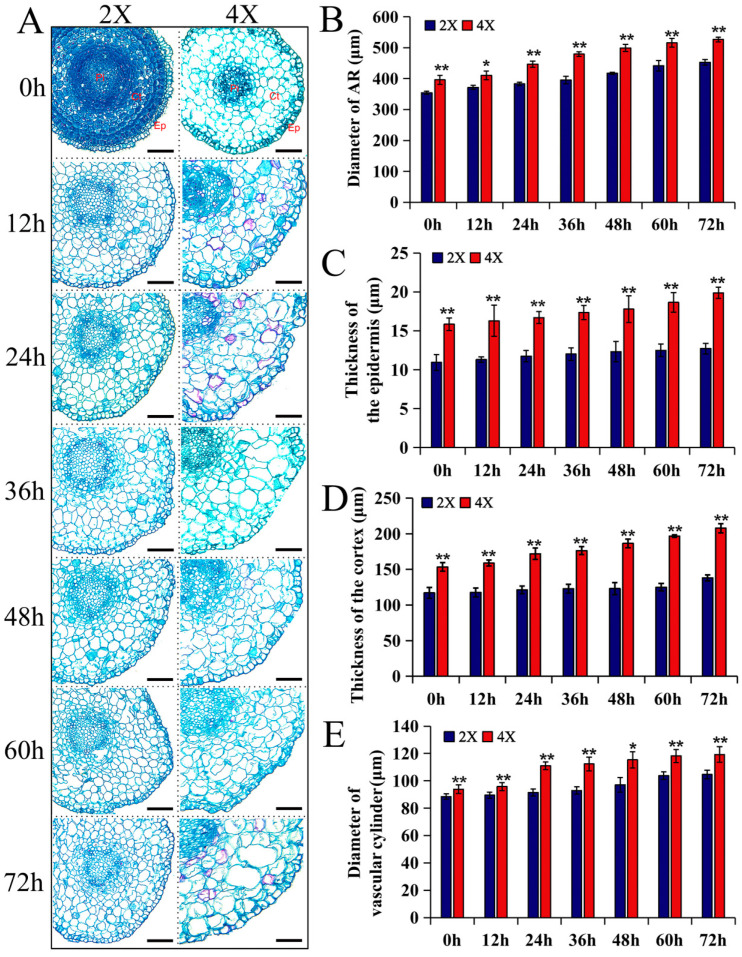
A comparative analysis of cross-sectional tissue thickness at the root tip between diploid and tetraploid poplars. (**A**) The cross-sectional structure of the root tip during the AR development of 2X and 4X. Ep: epidermis, Ct: cortex, Pi: pith, bars = 60 μm. AR diameter (**B**), epidermis thickness (**C**), cortex thickness (**D**), and vascular cylinder diameter (**E**) during AR development of 2X, diploid, and 4X, tetraploid. Data are represented by mean ± SD. * *p* < 0.05, ** *p* < 0.01 (Student’s *t*-test).

Observing the cross-sectional structures of the diploid and tetraploid ARs’ root tips at different development time points showed that the diameter of the tetraploid roots was greater than that of the diploids, and the epidermal, cortical, and vascular cylinder cells were also larger than those of the diploids (Figure 3A,B). The epidermis and cortex thicknesses and the vascular cylinder diameter of the tetraploids were also significantly greater than those of the diploids at seven time points of AR growth (Figure 3C–E). The above results showed that the tetraploid ARs were thicker because they had larger cells and thicker tissues.

**Figure 4:**
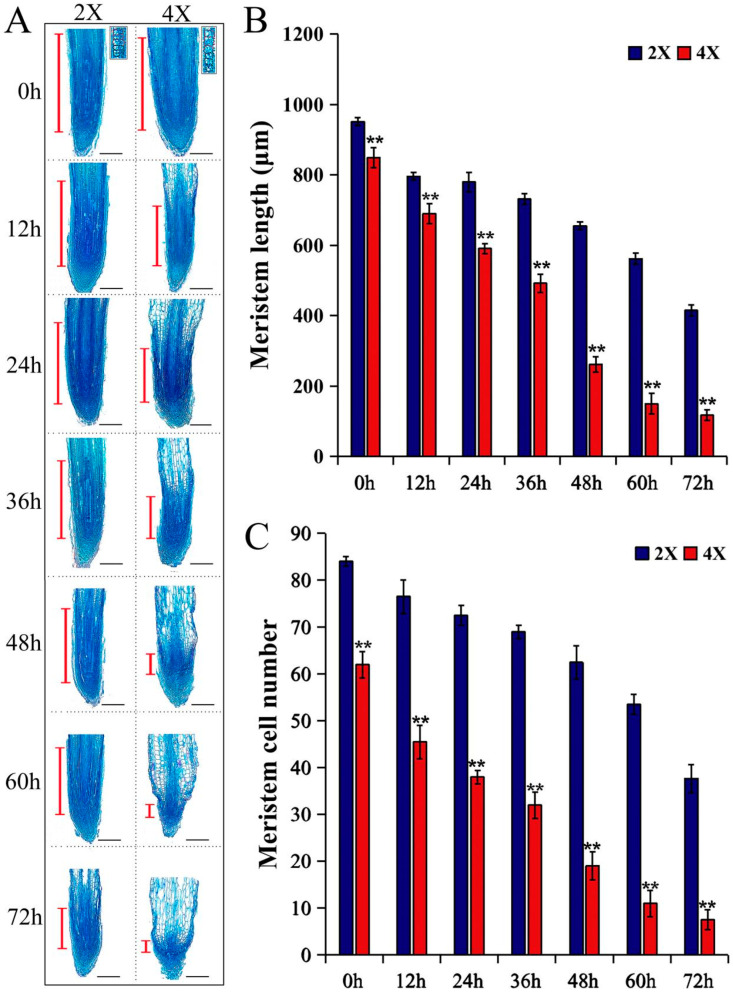
A comparative analysis of the longitudinal section meristem sizes of root tips between diploid and tetraploid poplars. (**A**) The longitudinal section structures of the root tips during AR development of 2X and 4X. The red line segment indicates the meristem length. The illustration in the upper right corner of 0 h shows the transition zone. Bar = 200 μm. (**B**) The meristem lengths and (**C**) meristem cell numbers of ARs at different time points. 2X, diploid; 4X, tetraploid. Data are represented by means ± SD. ** *p* < 0.01 (Student’s *t*-test).

The indeterminate growth and development of plant roots depends on the maintenance of the meristems located in the root tip [35]. The observation and comparison of the longitudinal structures of the AR tips at different development time points (Figure 4A) revealed that the lengths of the diploid and tetraploid meristems decreased gradually from 0 h to 72 h by 536.32 μm and 731.71 μm, respectively. The sharp decreases in AR meristem length in both varieties occurred during the 36 h to 48 h stages, which is consistent with the root length phenotype. In addition, the lengths of the tetraploid AR meristems were significantly shorter than those of the diploids at all seven time points from 0 h to 72 h (Figure 4B), and there were fewer meristem cells in the tetraploids than in the diploids (Figure 4C). All of the above results suggest that the size of the meristem, which maintains the growth of the ARs, is smaller in tetraploids than it is in diploids.

### 2.4. Changes in HRM Contents during AR Development

We detected thirty-seven HRMs in ARs, including nine auxins, namely IAA, IAA-Glu, MEIAA, ILA, IAA-Asp, OxIAA, ICAld, IAA-Ala, and IBA; sixteen CKs, namely cZ, mT9G, iP7G, tZOG, DHZ7G, cZ9G, cZROG, BAPR, K, BAP, tZ, KR, IPR, cZR, tZR, and pTR; five jasmonic acids (JAs), namely JA, JA-Phe, H2JA, JA-Val, and JA-ILE; two SAs, namely SA and SAG; two ABAs and an ABA derivative (hereafter referred to as abscisates (ABAs)), namely ABA and ABA-GE; one ethylene (ETH) precursor, namely ACC; one GA, namely GA19; and one strigolactone (SL), namely 5DS (Table S1).

**Figure 5:**
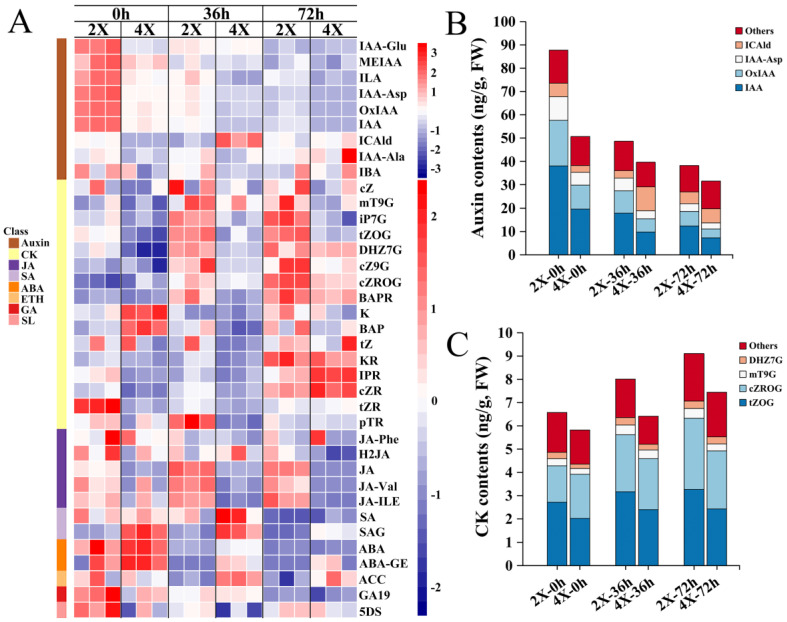
HRM contents during AR development in diploid and tetraploid poplars. (**A**) A heat map of the contents of HRMs during AR development. Red and blue colors indicate higher and lower relative levels, respectively. The color scale is shown on the right. Also shown are the HRMs differentially accumulated in different IAA types/forms (**B**) and CK types/forms (**C**) in the ARs. 2X, diploid; 4X, tetraploid.

The contents of certain HRMs in AR were significantly different at different ploidy or different development time points, as shown in the heat map of the relative abundance of HRMs (Figure 5A). Certain trends in specific HRMs’ content changes were observed throughout diploid and tetraploid AR development. The contents of most of the auxins (MEIAA, ILA, IAA-Asp, OxIAA, IAA, and total auxins), SAs (SA, and total SAs), ABAs (ABA, ABA-GE, and total ABAs), and GA19 decreased significantly with AR development; conversely, most of the CK contents (tZOG, DHZ7G, cZ9G, cZROG, BAPR, KR, cZR, and total CKs) increased significantly (Figure 5A, Table S1).

From 0 h to 72 h of AR growth, there was an antagonistic relationship between the total auxin contents (from 87.75 to 38.31 ng/g in diploids and from 49.37 to 31.59 ng/g in tetraploids with AR development) and the total CK contents (from 6.58 to 9.10 ng/g in diploids and from 5.81 to 7.44 ng/g in tetraploids with AR development) (Table S1). Furthermore, the predominant auxin types, namely IAA, OxIAA, and IAA-Asp (Figure 5B), and the CKs, namely tZOG and cZROG (Figure 5C), were consistent with the changes in the total auxin and CK contents, respectively. This finding supports the previous reports that auxin and CK act antagonistically to regulate root development [36]. Among the tested GAs (GA4, GA7, GA15, GA19, and GA24), GA19 was the predominant metabolite in the ARs, while the others were present in very low concentrations or not at all, indicating the specificity of GA19 during AR development.

**Figure 6:**
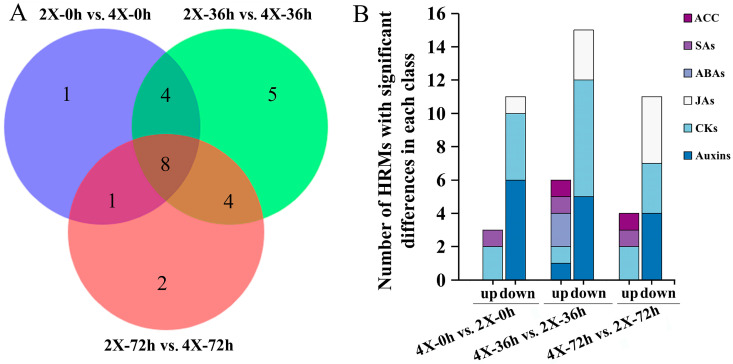
Analysis of differential HRM contents during the AR development of diploid and tetraploid poplars. (**A**) A Venn diagram showing the number of HRMs with significant differences (*p* < 0.05 for *t*-test) during AR development between diploids and tetraploids. (**B**) The number of significant up- and down-regulations in each class of HRMs in 4X vs. 2X AR development. Up, a significant increase in content; down, a significant decrease in content. CKs, cytokinins; JAs, jasmonic acids; ABAs, abscisates; SA, salicylic acids. 2X, diploid; 4X, tetraploid.

The following HRM content comparison groups—tetraploids for 0 h vs. diploids for 0 h; tetraploids for 36 h vs. diploids for 36 h; and tetraploids for 72 h vs. diploids for 72 h—had 14, 21, and 15 HRMs with significant differences in content, respectively (Figure 6A, Table S2). The comparison of HRM contents showed that most of the auxins (ILA, IAA-Asp, OxIAA, IAA, and total auxins), CKs (tZOG and total CKs), and JAs (JA and total JAs) were significantly lower in the tetraploids vs. the diploids, whereas the SA contents (SAG and total SAs) were considerably higher. Significant changes in the contents of other classes of HRMs were observed at specific development time points; the content of ACC was considerably higher in the tetraploids’ ARs than in those of the diploids, except at 0 h, where there was no significant difference; the ABA contents were also significantly higher in the tetraploid ARs at 36 h (Figure 6B, Table S3). Furthermore, except for the total SAs and total ABAs at 72 h of AR development, the magnitude of change in the contents of all eight classes of HRMs was greater in the diploids than it was in the tetraploids during AR development (Table S4).

### 2.5. Expression Analysis of Genes Related to Hormonal Signaling during AR Development

**Figure 7:**
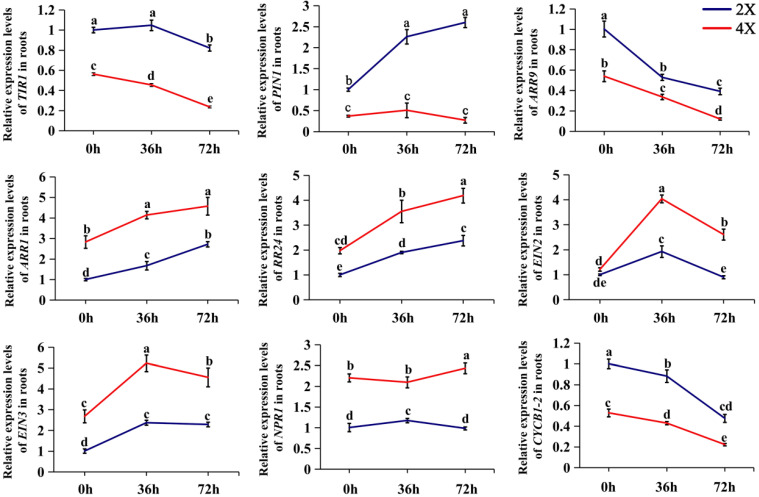
qPCR expression analyses of hormonal signaling genes in diploid and tetraploid poplars. The relative expression levels of *TIR1*, *PIN1*, *ARR9*, *ARR1*, *RR24*, *EIN2*, *EIN3*, *NPR1*, and *CYCB1-2* in the ARs of 2X and 4X were detected using qPCR. The *actin* gene was used as an internal control. The data represent at least three biological replicates. 2X, diploid; 4X, tetraploid. Data are represented by means ± SD. Different lowercase letters indicate significant differences between different samples, as determined by Duncan’s test (*p* < 0.05).

To further investigate the involvement of phytohormone-related genes in the AR development of diploids and tetraploids, we analyzed the temporal expression patterns of genes associated with hormonal signaling using qPCR (Figure 7). The results showed that the expression of the genes related to auxin (*TIR1* and *PIN1*) and CK (*ARR9*) signaling was lower in the tetraploids than it was in the diploids, while that related to ETH (*EIN2*) and SA (*NPR1*) signaling was higher in the tetraploids than in the diploids during AR development. The analysis of gene relative expression levels suggests that the signaling capacities of auxins and CKs in tetraploid ARs may be weaker than that in the diploids, while the signaling capacities of ETH and SA in the tetraploids may be stronger than that in the diploids. The relative expression levels of the cell cycle protein gene *CYCB1-2* were further examined, and the results showed that the expression of the *CYCB1-2* gene in the tetraploids was lower than that in the diploids throughout AR development. The relative quantification of the above genes suggests that differences in hormonal signaling and mitosis during AR development may contribute to the differences in AR traits between diploids and tetraploids.

## 3. Discussion

### 3.1. Variation in AR Morphology of Tetraploid Poplars

In this study, we investigated the dynamic changes in AR length (Figure 1C,E) and meristem size (Figure 3A,B) of hybrid poplars at seven time points during AR development. Compared to the diploids, the ARs of tetraploid hybrid poplars had a shorter meristem and grew significantly slower at each time point, ultimately displaying a shorter AR phenotype. These morphological observations are consistent with the previous findings on some herbs and fruit and timber trees [20,21,37]. However, some studies suggest that genome doubling promotes root growth. For example, tetraploid *Liriodendron sino-americanum* displayed more root growth than its diploid counterpart did [30]. Genome duplication in the tetraploid energy willow *Salix viminalis* L. significantly stimulated root growth, creating enlarged root systems for more efficient access to and extraction of nutrients and water [29]. Similarly, the length, mean diameter, surface area, and volume of the roots of tetraploid rootstock-grafted watermelon *Citrullus lanatus* were higher than those of diploid rootstock-grafted watermelon [18]. These differences in the root growth performance of plants after genome doubling may be due to the differences in species and environmental conditions.

The effect of genome doubling on root formation, such as its timing, the number of roots, and the rate of rooting, also varies among different species. Tetraploid *Echinacea purpurea* L. has demonstrated delayed root emergence and the lowest rooting rate compared to the haploid and diploid shoots, and tetraploids required higher concentrations of NAA for the earlier and better initiation of rooting [38]. During the nursery stage, grapevines grafted on two diploid ploidy rootstocks showed a significantly greater survival rate and more root growth than those on the corresponding tetraploid rootstocks [39]. In this study, there were no significant differences between diploid and tetraploid hybrid poplars in terms of the time of AR formation (Figure 2A), the number of ARs (Figure 2C), or the rooting rate (Figure 2B). The rooting capacities of both the diploid and tetraploid groups also did not differ in any of the three species of *E. rubra*, *E. rosea*, and *E. illinita* after 5 weeks [40]. Thus, whether the rooting ability of tetraploids is altered varies from species to species.

In addition, we observed that most diploid and tetraploid ARs almost stopped elongating after the last time point, expressing a gradual reddening of the root tip that lasted for some time, and then resumed elongation (Figure 1D). Studying the physiological, biochemical, and molecular bases behind this phenomenon in the future will be of great significance for improving root growth vitality.

### 3.2. HRMs Content Changes during Tetraploid AR Development

Auxin is a phytohormone that is a key regulator of root development [41,42]. Other plant hormones can affect the biosynthesis, transport, and signaling of auxin associated with root development. It is now widely accepted that CK interacts antagonistically with auxin to maintain RAM and ensure normal root growth and development [36]. In this study, whether diploid or tetraploid, the change trends of auxin and CK during AR development were opposite, which is consistent with this rule. In addition, the homeostasis between phytohormones is an important prerequisite for regulating plant growth, and a high IAA/CK ratio induced root development [43,44]. In this study, in addition to the auxin and CK contents in diploid ARs both being higher than those in tetraploids, the ratio of total auxins/total CKs in diploids (13.34) was also higher than that in tetraploids (8.50) at 0 h of AR development (Table S5); the ratios at other developmental time points were almost the same. Thus, although CKs decreased the size of the meristem by accelerating cell differentiation [41], the rate of auxin-promoting cell proliferation was much higher than the rate of CK-promoting cell differentiation in the diploid ARs compared to the tetraploid ARs during the early stages of AR development. This may be one of the reasons why the meristems of the diploid ARs were larger than those of the tetraploid ARs, thus promoting AR elongation growth.

In addition, some studies have shown that auxin–ETH crosstalk occurs in root growth [45]. ETH has long been thought to inhibit the growth of *Arabidopsis* roots by inhibiting root cell elongation without affecting RAM activity [46,47]. According to a recent report [48,49], ETH induced cell differentiation in the RAM through a typical ethylene response pathway, thereby negatively regulating the meristem size. ACC (1-Aminocyclopropanecarboxylic acid) is a direct precursor of ETH biosynthesis [50]. Therefore, in this study, the smaller root meristems of the tetraploids may also be caused by the synthesis of more ETH from the high content of ACC at two time points (36 h and 72 h) of the tetraploid ARs’ development, ultimately leading to slower AR growth. ABA inhibits root elongation in synergy with ethylene [51]. ABA was also a negative regulator of the development of AR in tomatoes [52]. ABA is characterized by its mediation of root growth, with low concentrations promoting root growth and high concentrations inhibiting root growth [53]. The application of high concentrations of exogenous ABA, or ABA accumulation by abiotic stress, inhibits primary root growth in *Arabidopsis* [54]. The meristem may be responsible for the inhibitory effect of ABA on root growth. High concentrations of ABA can repress cell division in the apical meristem [55]. The size of the meristem of the root tip was reduced when ABA was applied to *Arabidopsis* [56]. Therefore, the slow growth of tetraploid ARs in this study may also be due to a smaller meristem resulting from higher contents of ABA at 36 h of development.

Although it is generally believed that JA has an inhibitory effect on root growth, it can promote root growth when its concentration is very low. The root growth rate after applying a low-concentration JA treatment was faster than that of the control [57]. A treatment with synthetic propyl dihydro jasmonate was applied in order to promote plant root growth [58]. The application of exogenous JA has also been shown to promote quiescent center cell division [59]. In this study, although the concentration of JA in the diploids was higher than that in the tetraploids, it may not have reached a concentration capable of inhibiting root growth. In addition, the effect of SA on root length is concentration-dependent [60]. Low concentrations of SA stimulate root growth, while high concentrations of SA hinder root growth [61,62]. The concentrations of SA that activate/inhibit root growth vary among different species [60,61,62,63]. Only a few studies have reported a decrease or increase in root length after an SA treatment [64,65]. Moreover, SA controls root growth by regulating apical meristem activity [37,66]. In a previous study, a 30 μM SA treatment reduced the number of cells expressing the cell division marker CYCB1;1 in the proximal meristem, explaining the shorter root length after SA treatment [66]. The tetraploid ARs showed higher SA contents, less JA, and a decreased *CYCB1-2* expression level compared to those of the diploids in this study, confirming the previous research.

The qPCR results in this study illustrate the obvious differential expression of related genes during diploid and tetraploid AR development and further confirm the impact of complex hormonal crosstalk on the development of tetraploid ARs. For example, CKs modify the expression of the *PIN1* and *PIN7* genes, which regulate the cell-to-cell transport of auxin in roots, thereby affecting the auxin levels in root cells [67]. In this study, the expression level of *PIN1* (Figure 7) was consistently higher than that of the tetraploids during the development of diploid ARs, which may indicate that auxin in diploid ARs is better transported to different tissues within the roots to regulate cell division and differentiation, thus promoting root development. Epigenetic changes are an important source of transcriptional variation after polyploidization. Research has suggested that the expression levels of some miRNAs are higher in tetraploid apples (*Malus* × *domestica*) [68], *Paulownia tomentosa* [69], and *Lycium ruthenicum* [70] than in diploids, negatively regulating the expression levels of downstream target genes to control trait variation. Therefore, it is speculated that the slow growth of tetraploid ARs in this study may be linked to the up-regulation or down-regulation of miRNAs. It is crucial to investigate the changes in the expression of miRNAs and their target genes during tetraploid AR development in the future.

In this study, we observed the morphological, cytological, and HRM content differences during the development of tetraploid and diploid ARs and detected the differences in their hormone signaling-related genes using qPCR. The results showed that the slow growth of tetraploid ARs may be affected by the changes in various HRMs’ contents—mainly low contents of auxins (ILA, IAA-Asp, OxIAA, IAA, and total auxins), CKs (tZOG, cZR, and total CKs), and JAs (JA and total JAs); a low ratio of total auxins/total CKs at 0 h of AR development; and high contents of SAs (SAG and total SAs), which led to the smaller of the meristems in the tetraploids. The results of this study provide a basis for future investigations into the mechanism behind the slow development of tetraploid ARs in forest trees.

## 4. Materials and Methods

### 4.1. Plant Materials and Growth Conditions

The tissue culture seedlings of diploid hybrid poplars (*Populus simonii* × *P. nigra* var. *italica*) and corresponding autotetraploid hybrid poplars were used as research materials. Apical micro-cuttings (ca. 2–3 cm) with only 2–3 upper leaves were excised from 4-week-old in vitro plants and cultured on half-strength Murashige and Skoog medium (pH = 5.8) supplemented with 30 g/L sucrose and 2.5 g/L phytagel without any hormones at 25 °C, with a 16/8 h photoperiod.

### 4.2. AR Phenotypic Analysis

AR formation and growth were observed, and 3 biological replicates were formed separately, each containing 15 stem segments with an apical bud. The ratio of the number of stem segments forming roots to the total number of cultured stem segments represents the rooting rate. The rooting capacity was evaluated by calculating the percentage of the number of stem segments with different numbers of roots to the total number of stem segments. The length on the third day of root formation was recorded as 0 h root length, and then the root length was recorded every 12 h by dotting the root tip, and the root length was counted at 7 time points (0 h, 12 h, 24 h, 36 h, 48 h, 60 h, and 72 h). The ARL was measured with a ruler.

### 4.3. Anatomical Analysis

We collected three 1 cm root tips of ARs at each time point (0 h–72 h), fixed them with FAA (50% ethanol, 5% glacial acetic, and 4% formaldehyde), and then treated them using the method described in a previous study [71], which involved staining them with 1% Safranin O and 0.1% fast green. All the materials were observed under a biomicroscope (E100, Eclipse, Nikon, Tokyo, Japan) and photographed by an image acquisition and analysis system (DS-U3, Nikon). Cross-sections were made at a distance of about 0.5 cm from the root tip. Measurements of the root meristem size, root and vascular cylinder diameters, and epidermis and cortex thicknesses were taken and statistically analyzed. The meristem size was determined as the number of cells between the quiescent center and the first elongated cortex cell in the transition zone [35,72]. Three biological replicates were obtained. All the measurements were performed using ImageJ version 1.53 software.

### 4.4. Measurement of Plant HRMs Contents

At least 80–100 ARs were collected for each biological replicate at 0 h, 36 h, and 72 h. The three biological replicates were immediately frozen in liquid nitrogen and stored at −80 °C for the detection of HRMs. The extraction and purification of plant HRMs was performed using MetWare (http://www.metware.cn/) based on the AB Sciex QTRAP 6500 LC-MS/MS platform. The root samples were dissolved in 1 mL methanol/water/formic acid (15:4:1, *v*/*v*/*v*). A total of 10 μL of internal standard mixed solution (100 ng/mL) was added into the extract as an internal standard (IS) for quantification. The sample extracts were analyzed using a UPLC-ESI-MS/MS system (UPLC ExionLCTMAD; MS Applied Biosystems 6500 Triple Quadrupole). The HRMs were analyzed using scheduled multiple reaction monitoring (MRM).

### 4.5. RNA Isolation, cDNA Synthesis, and Quantitative Real-Time PCR (qPCR) Analysis

The total RNA of all the ARs was isolated using an RNA Easy Fast Plant Tissue Kit (DP452, TIANGEN Biotech (Beijing) Co., Ltd., Beijing, China), according to the manufacturer’s instructions. The quality of all the RNA samples was examined by performing agarose gel electrophoresis and examining the A260/280 wavelength ratio with a NanoDrop 8000 (Thermo Fisher Scientific CN, Shanghai, China). The PrimeScript RT Master Mix (Perfect Real Time) (Code No. RR036A; TaKaRa Biotech, Beijing, China) was used for cDNA synthesis. Hormone signaling plays a key role in root development [73], so the key genes in each hormone signaling pathway were selected for relative expression detection. The relative quantitative expression levels of the *TIR1*, *PIN1*, *ARR9*, *ARR1*, *RR24*, *EIN2*, *EIN3*, *NPR1*, and *CYCB1-2* genes were determined using an ABI QuantStudio 6 Flex Real-Time PCR system (Applied Biosystems, Foster City, CA, USA). The 10 μL reaction mixture was prepared, containing 5 μL NovoStart^®^ SYBR qPCR SuperMix Plus (Novoprotein Scientific, Inc., Shanghai, China), 0.8 μL primer pairs (Table S6) for the target gene, and a 1 μL cDNA template to perform qPCR. The actin gene (GenBank: EF418792.1) was amplified, with the primer pair ACTIN-FP and ACTIN-RP (Table S6) as the control. PCR was performed under the following conditions: denaturation at 95 °C for a minute, followed by 40 cycles of 95 °C for 20 s and 60 °C for a minute. Three biological replicates were made. The relative expression level was calculated using the 2^−ΔΔCt^ method. A sample diploid at 0 h served as the reference group, and the relative expressions of the other samples (diploid at 36 h; diploid at 72 h; tetraploid at 0 h; tetraploid at 36 h; and tetraploid at 72 h) were calculated.

### 4.6. Statistical Analyses

Statistical analyses were performed using Student’s *t*-test for pairwise comparisons between all the traits of the diploid and tetraploid samples. HRMs and qPCR data were subjected to analysis of variance (ANOVA) and Duncan’s new multiple range test (MRT) using SPSS version 25 (IBM, Inc., Armonk, NY, USA). *p* < 0.05 was considered to represent a significant difference. All the images were processed in Photoshop version 2023 (Adobe, Inc., San Jose, CA, USA).

## Data Availability

The data presented in this study are available on request from the authors. The raw data supporting the conclusions of this article will be made available by the authors on request.

## Supplementary Materials

The following supporting information can be downloaded at: https://www.mdpi.com/article/10.3390/plants13111430/s1. Table S1, Contents of HRMs in ARs of diploid and tetraploid poplar. Table S2, *p* value of *t*-test for HRMs content in 4X and 2X at the same time point of AR development. Table S3, HRMs of significant differences in the content of 4X compared to 2X during AR development. Table S4, Changes in the total content of different classes of HRMs during AR development. Table S5, The total auxins/total CKs ratio during AR development. Table S6, Sequences of gene-specific primers for qPCR detection.
