# Supplementary material for: The Slow Growth of Adventitious Roots in Tetraploid Hybrid Poplar (Populus simonii × P. nigra var. italica) May Be Caused by Endogenous Hormone-Mediated Meristem Shortening
Source: Plants (Basel). 2024 May 22;13(11):1430. doi: 10.3390/plants13111430 (PMC11174411; doi:10.3390/plants13111430)
Supplement: Supplementary file 1 [file plants-13-01430-s001.zip › plants-2984405-supplementary.pdf]

Table S1. Contents of HRMs in ARs of diploid and tetraploid poplar.

Classes	Hormones and Related Metabolites	2X-0h	2X-36h	2X-72h	4X-0h	4X-36h	4X-72h
Auxins	Indole-3-acetyl glutamic acid (IAA-Glu)	2.09 ± 0.08 a	1.31 ± 0.02 b	0.80 ± 0.05 d	1.04 ± 0.03 c	1.18 ± 0.04 b	0.82 ± 0.03 d
	Methyl indole-3-acetate (MEIAA)	0.50 ± 0.03 a	0.32 ± 0.03 c	0.29 ± 0.03 c	0.41 ± 0.01 b	0.31 ± 0.01 c	0.25 ± 0.03 c
	Indole-3-lactic acid (ILA)	1.73 ± 0.13 a	1.01 ± 0.12 b	0.70 ± 0.02 c	0.89 ± 0.05 bc	0.27 ± 0.07 d	0.32 ± 0.07 d
	Indole-3-acetyl-L-aspartic acid (IAA-Asp)	10.11 ± 0.2 a	5.43 ± 0.21 b	3.43 ± 0.17 c	5.44 ± 0.13 b	3.58 ± 0.01 c	2.66 ± 0.06 d
	oxindole-3-acetic acid (OxIAA)	19.61 ± 0.42 a	9.61 ± 0.51 b	6.16 ± 0.45 c	10.34 ± 0.37 b	5.68 ± 0.24 c	3.79 ± 0.01 d
	Indole-3-acetic acid (IAA)	38.12 ± 0.73 a	17.84 ± 0.98 b	12.35 ± 0.52 c	19.52 ± 0.31 b	9.65 ± 0.17 d	7.20 ± 0.04 e
	Indole-3-carboxaldehyde (ICAld)	5.70 ± 0.21 b	3.24 ± 0.50 c	4.99 ± 0.46 b	2.92 ± 0.08 c	10.16 ± 0.82 a	6.10 ± 0.41 b
	(3-Indolylacetyl)-L-alanine (IAA-Ala)	1.59 ± 0.12 ab	1.69 ± 0.15 ab	1.59 ± 0.08 ab	1.16 ± 0.11 b	1.27 ± 0.22 ab	2.31 ± 0.69 a
	Indole-3-butyric acid (IBA)	8.30 ± 0.35 a	8.16 ± 0.20 a	7.99 ± 0.37 a	7.64 ± 0.37 a	7.55 ± 0.05 a	8.13 ± 0.32 a
	Total auxins	87.75 ± 1.50 a	48.61 ± 2.10 b	38.31 ± 1.31 c	49.37 ± 0.54 b	39.65 ± 0.69 c	31.59 ± 1.37 d
Cytokinins (CKs)	cis-Zeatin (cZ)	0.01 ± 0.01 a	0.02 ± 0.01 a	0.02 ± 0.01 a	0.00 ± 0.00 a	0.01 ± 0.00 a	0.01 ± 0.01 a
	meta-Topolin-9-glucoside (mT9G)	0.30 ± 0.03 bc	0.41 ± 0.04 a	0.41 ± 0.04 a	0.26 ± 0.02 c	0.37 ± 0.02 ab	0.31 ± 0.03 abc
	N6-Isopentenyl-adenine-7-glucoside (iP7G)	0.22 ± 0.01 b	0.29 ± 0.01 a	0.32 ± 0.01 a	0.17 ± 0.01 b	0.20 ± 0.01 bc	0.18 ± 0.02 bc
	trans-Zeatin-O-glucoside (tZOG)	2.71 ± 0.04 b	3.17 ± 0.05 a	3.26 ± 0.06 a	2.02 ± 0.09 d	2.40 ± 0.12 c	2.43 ± 0.03 c
	Dihydrozeatin-7-glucoside (DHZ7G)	0.27 ± 0.01 b	0.31 ± 0.00 a	0.31 ± 0.02 a	0.19 ± 0.01 c	0.25 ± 0.00 b	0.30 ± 0.00 a
	cis-Zeatin-9-glucoside (cZ9G)	0.14 ± 0.00 bc	0.19 ± 0.01 a	0.20 ± 0.02 a	0.13 ± 0.01 c	0.16 ± 0.00 bc	0.17 ± 0.00 ab
	cis-Zeatin-O-glucoside riboside (cZROG)	1.57 ± 0.07 e	2.45 ± 0.10 bc	3.07 ± 0.06 a	1.89 ± 0.16 d	2.19 ± 0.04 c	2.49 ± 0.02 b
	Benzyladenosine (BAPR)	0.04 ± 0.00 b	0.07 ± 0.01 a	0.08 ± 0.00 a	0.04 ± 0.00 b	0.04 ± 0.00 b	0.07 ± 0.00 a

	Kinetin (K)	0.19 ± 0.01 bc	0.15 ± 0.02 c	0.23 ± 0.03 b	0.31 ± 0.01 a	0.14 ± 0.01 c	0.18 ± 0.03 bc
	Benzyladenine (BAP)	0.15 ± 0.01 cd	0.18 ± 0.01 bc	0.20 ± 0.02 b	0.24 ± 0.01 a	0.12 ± 0.01 d	0.17 ± 0.01 bc
	trans-Zeatin (tZ)	0.04 ± 0.01 a	0.04 ± 0.01 a	0.04 ± 0.01 a	0.03 ± 0.02 a	0.01 ± 0.00 a	0.04 ± 0.02 a
	Kinetin riboside (KR)	0.08 ± 0.01 b	0.07 ± 0.01 b	0.18 ± 0.02 a	0.05 ± 0.00 b	0.04 ± 0.01 b	0.16 ± 0.01 a
	N6-isopentenyladenosine (IPR)	0.26 ± 0.02 b	0.20 ± 0.02 c	0.28 ± 0.02 b	0.17 ± 0.01 c	0.17 ± 0.02 c	0.39 ± 0.00 a
	cis-Zeatin riboside (cZR)	0.19 ± 0.01 c	0.20 ± 0.01 c	0.27 ± 0.01 b	0.13 ± 0.00 e	0.16 ± 0.01 d	0.33 ± 0.01 a
	trans-Zeatin riboside (tZR)	0.31 ± 0.01 a	0.14 ± 0.01 b	0.14 ± 0.01 b	0.10 ± 0.01 c	0.10 ± 0.00 c	0.15 ± 0.01 b
	para-Topolin riboside (pTR)	0.09 ± 0.00 b	0.12 ± 0.01 a	0.07 ± 0.00 b	0.07 ± 0.01 b	0.05 ± 0.00 c	0.07 ± 0.00 b
	Total CKs	6.58 ± 0.04 d	8.00 ± 0.20 b	9.10 ± 0.18 a	5.81 ± 0.21 e	6.41 ± 0.15 d	7.44 ± 0.11 c
Jasmonates (JAs)	[(-)-Jasmonoyl]-(l)-phenalanine (JA-Phe)	0.17 ± 0.05 a	0.12 ± 0.02 a	0.11 ± 0.02 a	0.15 ± 0.03 a	0.09 ± 0.01 a	0.13 ± 0.05 a
	Dihydrojasmonic acid (H2JA)	0.48 ± 0.05 a	0.39 ± 0.03 ab	0.40 ± 0.03 a	0.39 ± 0.04 ab	0.43 ± 0.05 a	0.27 ± 0.02 b
	Jasmonic acid (JA)	19.91 ± 0.36 c	29.66 ± 0.69 a	27.38 ± 0.43 b	10.95 ± 0.87 d	11.88 ± 0.28 d	9.29 ± 0.29 e
	[(-)-Jasmonoyl]-(L)-valine (JA-Val)	0.10 ± 0.01 a	0.12 ± 0.00 a	0.10 ± 0.01 a	0.06 ± 0.02 b	0.03 ± 0.01 b	0.03 ± 0.00 b
	Jasmonoyl-L-isoleucine (JA-ILE)	1.58 ± 0.09 a	1.78 ± 0.04 a	1.90 ± 0.13 a	1.12 ± 0.27 b	0.43 ± 0.03 c	0.43 ± 0.03 c
	Total JAs	22.24 ± 0.54 c	32.07 ± 0.77 a	29.89 ± 0.57 b	12.67 ± 1.17 d	12.85 ± 0.34 d	10.15 ± 0.31 e
Salicylates (SAs)	Salicylic acid (SA)	115.96 ± 10.59 ab	103.96 ± 11.89 bc	66.32 ± 1.45 d	114.32 ± 5.34 ab	141.48 ± 17.55 a	74.99 ± 5.48 cd
	Salicylic acid 2-O-β-glucoside (SAG)	577.34 ± 21.49 c	540.05 ± 33.53 c	447.14 ± 6.65 c	1145.13 ± 51.61 a	1091.12 ± 84.62 a	737.24 ± 23.42 b
	Total SAs	693.30 ± 23.74 b	644.01 ± 45.19 bc	513.46 ± 6.73 c	1259.45± 56.89 a	1232.60 ± 102.02 a	812.23 ± 19.83 b
Abscisates (ABAs)	Abscisic acid (ABA)	4.98 ± 0.67 a	2.18 ± 0.07 bc	1.84 ± 0.05 c	5.45 ± 0.21 a	3.01 ± 0.08 b	1.74 ± 0.06 c
	ABA-glucosyl ester (ABA-GE)	14.27 ± 7.44 ab	0.00 ± 0.00 c	0.00 ± 0.00 c	25.60 ± 1.99 a	12.13 ± 2.20 bc	8.69 ± 4.41 bc

	Total ABAs	19.24 ± 7.98 ab	2.18 ± 0.66 c	1.84 ± 0.05 c	31.05 ± 2.19 a	15.13 ± 2.16 b	10.43 ± 4.39 bc
Ethylene (ETH)	1-Aminocyclopropanecarboxylic acid (ACC)	93.41 ± 6.89 a	79.38 ± 1.74 b	77.18 ± 4.05 b	89.86 ± 3.10 ab	102.37 ± 1.51 a	96.24 ± 3.87 a
Gibberellin (GA)	Gibberellin A19 (GA19)	2.69 ± 0.27 a	1.68 ± 0.11 b	0.99 ± 0.03 c	1.77 ± 0.25 b	1.69 ± 0.03 b	0.80 ± 0.17 c
Strigolactone (SL)	Deoxystrigol (5DS)	2.66 ± 0.16 a	2.02 ± 0.07 bc	2.13 ± 0.12 ab	1.77 ± 0.28 bc	1.44 ± 0.24 c	1.99 ± 0.12 bc

Notes: The table listed the HRMs and the summation of all individual HRMs detected in each class. The values were means of three independent samples (n = 3) as ng/g FW. Data were expressed as means ± SE. Different lower-case letters beside the values indicate significant differences in the content of HRMs between samples ($P < 0.05$). Total auxins, CKs, JAs, SAs, and ABAs were the summation of all individual HRMs detected in each class. 2X, diploid. 4X, tetraploid.

Table S2. ***P*** **value of** ***t*****-test for HRMs content in 4X and 2X at the same time point of AR development.**

Classes	Hormones and Related Metabolites	4X-0h vs 2X-0h	4X-36h vs 2X-36h	4X-72h vs 2X-72h
Auxins	Indole-3-acetyl glutamic acid (IAA-Glu)	0.0002	0.0464	0.8158
	Methyl indole-3-acetate (MEIAA)	0.0625	0.7371	0.4109
	Indole-3-lactic acid (ILA)	0.0043	0.0064	0.0063
	Indole-3-acetyl-L-aspartic acid (IAA-Asp)	0.0000	0.0008	0.0127
	oxindole-3-acetic acid (OxIAA)	0.0001	0.0023	0.0063
	Indole-3-acetic acid (IAA)	0.0000	0.0012	0.0006
	Indole-3-carboxaldehyde (ICAld)	0.0003	0.0020	0.1485
	(3-Indolylacetyl)-L-alanine (IAA-Ala)	0.0622	0.1826	0.3583
	Indole-3-butyric acid (IBA)	0.2609	0.0413	0.7931
	Total auxins	0.0000	0.0154	0.0241
Cytokinins (CKs)	cis-Zeatin (cZ)	0.3498	0.3206	0.2398
	meta-Topolin-9-glucoside (mT9G)	0.2489	0.4386	0.0965
	N6-Isopentenyl-adenine-7-glucoside (iP7G)	0.0592	0.0034	0.0027
	trans-Zeatin-O-glucoside (tZOG)	0.0020	0.0048	0.0002
	Dihydrozeatin-7-glucoside (DHZ7G)	0.2489	0.4386	0.0965
	cis-Zeatin-9-glucoside (cZ9G)	0.8354	0.4783	0.0670
	cis-Zeatin-O-glucoside riboside (cZROG)	0.1474	0.0708	0.0010
	Benzyladenosine (BAPR)	0.3071	0.0088	0.0925
	Kinetin (K)	0.0030	0.6395	0.3159
	Benzyladenine (BAP)	0.0005	0.0168	0.3159
	trans-Zeatin (tZ)	0.7602	0.1184	0.9077
	Kinetin riboside (KR)	0.0523	0.0718	0.3265
	N6-isopentenyladenosine (IPR)	0.0062	0.3433	0.0033
	cis-Zeatin riboside (cZR)	0.0052	0.0232	0.0305
	trans-Zeatin riboside (tZR)	0.0001	0.0080	0.5304
	para-Topolin riboside (pTR)	0.2397	0.0008	0.7407
	Total CKs	0.0239	0.0033	0.0014
Jasmonates (JAs)	[(-)-Jasmonoyl]-(l)-phenalanine (JA-Phe)	0.7534	0.2528	0.7696
	Dihydrojasmonic acid (H2JA)	0.2194	0.5304	0.0294
	Jasmonic acid (JA)	0.0007	0.0000	0.0000
	[(-)-Jasmonoyl]-(L)-valine (JA-Val)	0.2321	0.0002	0.0027
	Jasmonoyl-L-isoleucine (JA-ILE)	0.1806	0.0000	0.0004
	Total JAs	0.0017	0.0000	0.0000
Salicylates (SAs)	Salicylic acid (SA)	0.8968	0.1515	0.2012
	Salicylic acid 2-O-β-glucoside (SAG)	0.0005	0.0038	0.0003
	Total SAs	0.0008	0.0062	0.0001
Abscisates	Abscisic acid (ABA)	0.5373	0.0012	0.2878

(ABAs)	ABA-glucosyl ester (ABA-GE)	0.2150	0.0314	0.1876
	Total ABAs	0.2267	0.0267	0.1897
Ethylene (ETH)	1-Aminocyclopropanecarboxylic acid (ACC)	0.6631	0.0006	0.0273
Gibberellin (GA)	Gibberellin A19 (GA19)	0.0683	0.9389	0.3375
Strigolactone (SL)	Deoxystrigol (5DS)	0.0519	0.0849	0.4416

Notes: HRMs, hormones and related metabolites. 2X, diploid. 4X, tetraploid.

Table S3. HRMs of significant differences in the content in 4X compared to 2X during AR development.

Comparison group	UP	Down
4X-0h vs 2X-0h	Kinetin (K)	Indole-3-acetyl glutamic acid (IAA-Glu)
	Benzyladenine (BAP)	Indole-3-lactic acid(ILA)
	Salicylic acid 2-O-β-glucoside (SAG)	Indole-3-acetyl-L-aspartic acid (IAA-Asp)
		oxindole-3-acetic acid(OxIAA)
		Indole-3-acetic acid(IAA)
		Indole-3-carboxaldehyde (ICAld)
		trans-Zeatin-O-glucoside (tZOG)
		N6-isopentenyladenosine (IPR)
		cis-Zeatin riboside (cZR)
		trans-Zeatin riboside (tZR)
		Jasmonic acid (JA)
4X-36h vs 2X-36h	Indole-3-carboxaldehyde (ICAld)	Indole-3-acetyl glutamic acid (IAA-Glu)
	Indole-3-butyric acid (IBA)	Indole-3-lactic acid(ILA)
	Salicylic acid 2-O-β-glucoside (SAG)	Indole-3-acetyl-L-aspartic acid (IAA-Asp)
	Abscisic acid (ABA)	oxindole-3-acetic acid(OxIAA)
	ABA-glucosyl ester (ABA-GE)	Indole-3-acetic acid(IAA)
	1-Aminocyclopropanecarboxylic acid (ACC)	N6-Isopentenyl-adenine-7-glucoside (iP7G)
		trans-Zeatin-O-glucoside (tZOG)
		Benzyladenosine (BAPR)
		Benzyladenine (BAP)
		cis-Zeatin riboside (cZR)
		trans-Zeatin riboside (tZR)
		para-Topolin riboside (pTR)
		Jasmonic acid (JA)
		[(-)-Jasmonoyl]-(L)-valine (JA-Val)
		Jasmonoyl-L-isoleucine (JA-ILE)
4X-72h vs 2X-72h	N6-isopentenyladenosine (IPR)	Indole-3-lactic acid(ILA)
	cis-Zeatin riboside (cZR)	Indole-3-acetyl-L-aspartic acid (IAA-Asp)
	Salicylic acid 2-O-β-glucoside (SAG)	oxindole-3-acetic acid(OxIAA)
	1-Aminocyclopropanecarboxylic acid (ACC)	Indole-3-acetic acid(IAA)
		N6-Isopentenyl-adenine-7-glucoside (iP7G)
		trans-Zeatin-O-glucoside (tZOG)
		cis-Zeatin-O-glucoside riboside (cZROG)
		Dihydrojasmonic acid (H2JA)
		Jasmonic acid (JA)
		[(-)-Jasmonoyl]-(L)-valine (JA-Val)
		Jasmonoyl-L-isoleucine (JA-ILE)

Notes: HRMs, hormones and related metabolites. 2X, diploid. 4X, tetraploid. Up, the significant increase in contents. Down, the significant decrease in contents.

Table S4. Changes in the total content of different classes of HRMs during AR development.

Classes	2X		4X	
	C_{36h}-C_{0h}	C_{72h}-C_{0h}	C_{36h}-C_{0h}	C_{72h}-C_{0h}
Total Auxins	-39.14	-49.44	-9.72	-17.78
Total CKs	+1.42	+2.52	+0.6	+1.63
Total JAs	+9.83	+7.65	+0.18	-2.52
Total SAs	-49.29	-179.84	-26.85	-447.22
Total ABAs	-17.06	-17.4	-15.92	-20.62
ACC	-14.03	-16.23	+12.51	+6.38
GA19	-1.01	-1.7	-0.08	-0.97
5DS	-0.64	-0.53	-0.33	+0.22

Notes: HRMs: hormones and related metabolites. C, indicates hormone content. +, indicates an increase in HRMs content. -, indicates a decrease in HRMs content. 2X, diploid. 4X, tetraploid.

Table S5. The total auxins/total CKs ratio during AR development.

Different ploidy	total auxins/total CKs ratio		
	**0h**	**36h**	**72h**
2X	13.34	6.08	4.21
4X	8.50	6.19	4.25

Notes: 2X, diploid. 4X, tetraploid.

Table S6. Sequences of gene-specific primers for qPCR detection

Target gene	Primer	Sequence of gene-specific primer
TIR1	TIR1-FP	5’-CTGCCCTGATTTCACCCACT-3’
	TIR1-RP	5’-AGTGCAAGTCCTCACCACAG-3’
PIN1	PIN1-FP	5’-CCCAGAAGGTCGTGTGATCA-3’
	PIN1-RP	5’-CGCCACGTTTAGCAGAGCTA-3’
ARR9	ARR9-FP	5’-GATGTTTGGAGGAGGGAGCC-3’
	ARR9-RP	5’-GCTGCACTGGTTGTTTCTGC-3’
ARR1	ARR1-FP	5’-AGATGCAACGAGCCATGTGA-3’
	ARR1-RP	5’-GCTGTTGTTTGAGAAGCGCA-3’
RR24	RR24-FP	5’-CAGCAACCCAAAAGAAGCCC-3’
	RR24-RP	5’-TGGAGATGGCTTGCCACATT-3’
EIN2	EIN2-FP	5’-CGAGGCCCAACTTCTTCAGT-3’
	EIN2-RP	5’-TCAGATCCTCGTCAGCTCCA-3’
EIN3	EIN3-FP	5’-ATCCGCAAGCTTGTGAGACA-3’
	EIN3-RP	5’-ATGACAATGGTGGGCAGGAG-3’
NPR1	NPR1-FP	5’-AATGGGCCGACGATACTTCC-3’
	NPR1-RP	5’-TCATCTGGGGTGCCCTTTTC-3’
CYCB1-2	CYCB1-2-FP	5’-TCGGCCTCGATACCAAGAGA-3’
	CYCB1-2-RP	5’-CCATCTGAACGGGAAAGGCT-3’
ACTIN	ACTIN-FP	5’-TCATCGGAATGGAAGCTGCTGGTA-3’
	ACTIN-RP	5’-TAGTGGAACCACCACTGAGCACAA-3’
